# Gardeniae Fructus Attenuates Thioacetamide-Induced Liver Fibrosis in Mice via Both AMPK/SIRT1/NF-κB Pathway and Nrf2 Signaling

**DOI:** 10.3390/antiox10111837

**Published:** 2021-11-19

**Authors:** Mi-Rae Shin, Jin A Lee, Minju Kim, Sehui Lee, Minhyuck Oh, Jimin Moon, Joo-Won Nam, Hyukjae Choi, Yeun-Ja Mun, Seong-Soo Roh

**Affiliations:** 1 Department of Herbology, College of Korean Medicine, Daegu Haany University, Daegu 42158, Korea; with750@naver.com (M.-R.S.); tgs02022@naver.com (J.A.L.); mj8976@naver.com (M.K.); k18dw@naver.com (S.L.); gjo53@naver.com (M.O.); 2 College of Pharmacy, Yeungnam University, Gyeongsan 38541, Korea; hyp1112@yu.ac.kr (J.M.); jwnam@yu.ac.kr (J.-W.N.); h5choi@yu.ac.kr (H.C.); 3 Research Institute of Cell Culture, Yeungnam University, Gyeongsan 38541, Korea; 4 Department of Anatomy, School of Korean Medicine, Wonkwang University, Iksan 54538, Korea; yjmun@wku.ac.kr; 5 Research Center of Traditional Korean Medicine, Wonkwang University, Iksan 54538, Korea

**Keywords:** liver fibrosis, Gardeniae Fructus, thioacetamide, AMPK/SIRT1 pathway, Nrf2 signaling

## Abstract

Liver fibrosis, which means a sort of the excessive accumulation of extracellular matrices (ECMs) components through the liver tissue, is considered as tissue repair or wound-healing status. This pathological stage potentially leads to cirrhosis, if not controlled, it progressively results in hepatocellular carcinoma. Herein, we investigated the pharmacological properties and underlying mechanisms of Gardeniae Fructus (GF) against thioacetamide (TAA)-induced liver fibrosis of mice model. GF not only attenuated hepatic tissue oxidation but also improved hepatic inflammation. We further confirmed that GF led to ameliorating liver fibrosis by ECMs degradations. Regarding the possible underlying mechanism of GF, we observed GF regulated epigenetic regulator, Sirtuin 1 (SIRT1), in TAA-injected liver tissue. These alterations were well supported by SIRT1 related signaling pathways through regulations of its downstream proteins including, AMP-activated protein kinase (AMPK), p47^phox^, NADPH oxidase 2, nuclear factor erythroid 2–related factor 2 (Nrf2), and heme oxygenase-1, respectively. To validate the possible mechanism of GF, we used HepG2 cells with hydrogen peroxide treated oxidative stress and chronic exposure conditions via deteriorations of cellular SIRT1. Moreover, GF remarkably attenuated ECMs accumulations in transforming growth factor–β1-induced LX-2 cells relying on the SIRT1 existence. Taken together, GF attenuated liver fibrosis through AMPK/SIRT1 pathway as well as Nrf2 signaling cascades. Therefore, GF could be a clinical remedy for liver fibrosis patients in the future.

## 1. Introduction

Liver fibrosis is provoked by chronic hepatic diseases and results from a disordered wound healing response [1]. The pathophysiological feature of liver fibrosis has mainly been characterized by the deposition of excessive extracellular matrix (ECM) molecules, particularly various collagen types. In addition, advanced liver fibrosis could lead to portal hypertension, hepatic dysfunction, liver cirrhosis, liver failure, and sometimes require liver transplantation [2]. For that reason, understanding the underlying mechanism, accurate diagnosis, and systematic approach in the early stages will accelerate its treatment and lessen the prevalence of cirrhosis. Hepatic stellate cells (HSCs) were reported as the fundamental collagen-producing cell in the liver since the 1980s [3]. Key signaling that regulates HSCs’ fibrogenic actions was precisely described. Experimental animal models for studying liver fibrogenesis were developed and led to the identification of key fibrogenic mediators [4]. To induce liver fibrosis, many chemical inducers such as thioacetamide, chloroform, iodoform, and carbon tetrachloride have been widely used. Chronic and persistent treatment using TAA induces liver injury, fibrosis, and finally cirrhosis derived from activation of HSCs [5,6]. Activated HSCs are alpha-smooth muscle actin (α-SMA)-positive cells that produce ECMs protein, including Collagen I [7]. Therefore, various studies to investigate target factors of activated HSCs were considered as crucial progress in a reversal of liver fibrosis [8].

Gardeniae Fructus (GF, the dried ripe fruits of *Gardenia jasminoides* Ellis) not only has been popularly applied to traditional medicine to treat hepatic disorders or to decrease various inflammation but also epidemically used as an excellent natural colorant. A total of 162 components have been separated and identified from GF [9]. Among the various compounds from GF, geniposide is one of the most important iridoid compounds and it was validated the pharmacological properties including anti-oxidative, anti-inflammatory, hepatoprotective, anti-depressive, anxiolytic activities, and beneficial effects on cardiovascular and digestive systems either in vitro or in vivo [10,11]. 

On the other hand, Silymarin, which is used as a positive control in this experiment, is distributed in the fruit and seeds of milk thistle. Silymarin exerts the hepatoprotective effects from inflammation, oxidative stress, and consequent cytotoxicity in patients with fibrosis or cirrhosis without serious adverse events [12].

In the current study, as purposed to investigate the pharmacological properties of GF against TAA (i.p.) to induce liver fibrosis of mice model, and our data elucidated new knowledge about anti-hepatofibrotic effects of GF. Additionally, we also provide the possible mechanisms GF which could mainly affect AMPK/SIRT1 pathway and/or Nrf2 signaling cascades on the liver fibrosis as well as chronic TAA exposure hepatic oxidative stress conditions. 

## 2. Materials and Methods

### 2.1. Materials

Sodium carbonate (Cat No. 222321), quercetin (Cat No. Q4950), dimethyl sulfoxide (DMSO, D2650), silymarin (Cat No, S0292), and thioacetamide (Cat No. 163678) were provided by Sigma Aldrich Co. (St Louis, MO, USA). L-ascorbic acid was provided by Alfa Aesar (Cat No. A15613, Lancashire, UK). 

### 2.2. Preparation of the Plant Material and Finger Printing Analysis of GF

The GF used in this experiment was a medicinal herb produced in Goheung, Jellannam-do in 2019, and was supplied from Bonchowon (Yeongcheon, Gyeongsanbuk-do). Dried GF (100 g) boiled with water (1 L) at 25 °C for 2 h. The powder of GF was a yield of 24.6% by weight and kept at −80 °C before use. GF power (1 mg) was dissolved in 2 mL of 100% methanol. The solution was centrifuged at 13,500 rpm for 3 min and the supernatant was collected for component analysis. We injected 3 μL of the sample into a Waters Acquity UPLC system (Waters^®^, Milford, MA, USA) using a reversed-phase C18 column (Phenomenex HPLC part: Luna 3μ C18(2) 100 Å, 150 mm × 4.6, Torrance, CA, USA). The mobile phase composition was as follows: solvent A (5% acetonitrile in deionized water with 0.05% formic acid), solvent B (100% acetonitrile with 0.05% formic acid). The gradient conditions; A:B = 90:10 (0 min) → 90:10 (2 min) → 80:20 (3 min) → 80:20 (6 min) → 0:100 (8 min) → 0:100 (10 min) → 90:10 (11 min) → 90:10 (14 min). The flow rate was 0.7 mL/min with UV absorption monitoring at 254 nm. The peak of geniposide was assigned by comparison of retention time and UV spectrum of authentic standard. Geniposide was detected as a major compound from the chromatogram of the extract. Representative UPLC chromatogram was illustrated in Supplementary Figure S1A–D. Quantification of geniposide in the extract was performed by peak area measurement. The calibration curve of geniposide was made at the concentration of 6.25, 12.5, 25, 50, 100, 200, and 400 μg/mL with an injection volume of 3 μL each and the replication of five. The regression coefficient (*R^2^*) was calculated as 0.9994 (standard curve; y = 4.3285x − 8.4191). 

### 2.3. Mice Treatment

The animal protocol was approved by the Ethics Committee of the Daegu Haany University and performed according to ‘the Guidelines for Animal Experiment’. C57BL/6 mice (male, 20–25 g) were purchased from DBL (Eumseong, Korea). The mice were maintained at 22 ± 2 °C and controlled with a 12 h light/dark cycle and humidity (50 ± 5%). After 1 week of adaptation, a total of thirty-six mice were divided into 4 groups (*n* = 9 for each group): Normal, Control (TAA only), GF (200 mg/kg), and Silymarin (50 mg/kg), respectively. Mice in the normal group were intraperitoneally injected 0.9% normal saline orally administrated with distilled water (DW); the control group received TAA (i.p.) and DW (peroral, p.o.); the Silymarin group received TAA (i.p.) and Silymarin at 50 mg/kg/day (p.o.); the GF group received TAA (i.p.) and GF at 200 mg/kg/day (p.o.) for 8 weeks of experimental periods (Supplementary Figure S2). 

Liver fibrosis was induced by TAA three times injection per week for 8 weeks according to an escalating treatment dose protocol (100 mg/kg for 1st week; 200 mg/kg for from 2nd to 3rd week, and 400 mg/kg for 4th to 8th week, respectively). The treatment drugs for liver fibrosis both GF (200 mg/kg/day) and Silymarin (50 mg/kg/day) were continuously administrated for 8 weeks 90 min prior to TAA injection. On the final day of the experiment, whole blood samples were collected from the abdominal vein and centrifuged at 4000 rpm for 10 min (at 4 °C), then stored in −80 °C freezer for further analyses. Liver tissues were removed immediately transferred 10% neutral formalin for the purpose of histological analysis and liquid nitrogen gas then stored at −80 °C for further biochemistry analysis, respectively.

### 2.4. Histological Examination

Liver tissue samples were fixed in 10% formalin then processed for embedding and sectioning at the Kyeongbook National University Hospital BMRI (Daegu, Korea). Liver sections (3 μm thickness) were stained using hematoxylin and eosin (H&E), Masson’s trichrome (MT), and Sirius Red staining which were followed by the standard protocol. The images were captured using Olympus BX51 Microscope (Olympus Co., Ltd., Tokyo, Japan, 40-, 100-, and 200-× magnifications) and then analyzed using the I-Solution Lite software program (IMTechnology, Vancouver, BC, Canada). 

### 2.5. Analysis of Serum Biochemistry

Liver enzymes such as aspartate aminotransferase (AST) and alanine aminotransferase (ALT) were measured by serum levels using a Transaminase CⅡ-Test (Wako Pure Chemical Industries Ltd., Osaka, Japan). Serum ammonia was measured by ELISA kit (Abcam, Cambridge, UK) according to the manufacturer’s instructions.

### 2.6. Analysis of Immunohistochemistry (IHC) and Immunofluorescence (IF)

IHC analysis was performed for detecting Kupffer cells (F4/80), bile duct cells (CK-19), and Collagen type 3. We further completed IF analyses for observing 4-HNE, desmin, α-smooth muscle actin (α-SMA), and Collagen type 1, respectively (Supplementary Figure S7). Briefly, paraffin embedding liver tissue sections were deparaffinized and rehydrated with various gradations of ethanol. After washing with liver sections with tap water, they were progressed to peroxidase removal with 5% hydrogen peroxide in absolute methanol for 5 min. Tissues were incubated in 2.5% normal horse serum (NHS) then applied primary antibodies for overnight at 4 °C. Tissue sections were washed applied horseradish peroxidase (HRP)-conjugated secondary antibodies, then applied the signals using 3-Amino-9-ethyl carbazol (AEC) and mounted with mounting buffer. The images of IHC analysis were captured using light microscopy conditions (Olympus BX51, Tokyo, Japan, 100- and 400-×) and IF images were observed by fluorescent filters equipped microscope (Carl Zeiss, Germany, 100- and 200-×). The quantification analysis of each positive signal for IHC or IF image was obtained from the randomly selected sections of at least four fields of each sample using Image J 1.52 software (NIH, Bethesda, MD, USA). 

### 2.7. Measurement of Myeloperoxidase (MPO) and TBA-Reactive Substance (TBARS) Levels

MPO in serum was determined via a colorimetric kit of BioVision, Inc. (Milpitas, CA, USA). TBARS assay to evaluate malondialdehyde (MDA) was estimated according to the method of Mihara and Uchiyama [13].

### 2.8. Cell Cultures

For validating possible mechanisms of GF on hepatic oxidation and fibrosis we cultured human blastoma cell line, HepG2, and hepatic stellate cells (HSCs) line, LX-2 cells, respectively. HepG2 cells were cultured in 10% fetal bovine serum (FBS) containing DMEM, and LX-2 cells were cultured 5% FBS contained DMEM under the condition of 5% CO_2_ supplementary with 37 °C incubators, respectively.

For hepatic oxidative stress condition, we treated 500 µM of hydrogen peroxide (H_2_O_2_) during 6- and 24-h after pre-treatment with 50, 100, and 200 µg/mL of GF, then cells were washed with 10 mM PBS (pH 7.3) twice and fixed in 4% paraformaldehyde (PFA) or cell lysis buffers (for Western blot analysis) according to each specific experimental condition. 

For liver fibrosis condition, we active LX-2 cells by transforming growth factor (TGF)–β1 (10 ng/mL) for 18 h incubation after pre-treatment with GF (200 µg/mL) for 6 h and followed to the washing and fixing for further analysis, respectively. IHC or IF analysis using HepG2 cells or LX-2 cells, we used standard protocol. Briefly, the cells were washed with PBS (10 mM, pH 7.3) twice and fixed in 4% paraformaldehyde for 1 h at RT. Cells were washed with PBS twice and incubated with 0.3% Triton X-100 for 5 min at RT, then underwent PBS washing twice. Cells were treated 2.5% NHS which is known as blocking buffer for 1 h at RT and primary antibodies were treated then incubated at 4 °C overnight. After washing with PBS three times (each time for 10 min) at RT, we treated fluorescent conjugated secondary antibodies for IF analysis or HRP-conjugated secondary antibody for IHC, respectively.

For IF analysis, we followed the further washing steps using twice PBS and one 0.05% PBST for 10 min, and IHC analysis we washed with PBS three times using PBS, respectively. After washing secondary antibodies for each experiment, the positive signals were detected by the development of AEC and performed counterstaining using hematoxylin. In the case of IF analysis, we completed the counterstaining by application of DAPI solution to the cells. 

Images were captured according to each experiment type as followed by the liver tissue sections.

### 2.9. Western Blotting

To obtain cell samples, we used the lysis buffer was composed of various compounds as follows; 50 mM Hepes, pH 7.5, 150 mM NaCl, 10% glycerol, 1% Triton X-100, 1.5 mM MgCl_2_, 1 mM EGTA, 10 mM sodium pyrophosphate, 100 mM sodium fluoride, and freshly added 100 μM sodium vanadate, 1 mm PMSF, 10 μg/mL aprotinin, and 10 μg/mL leupeptin, respectively or NP-40 lysis buffer (1% NP-40, 20 mM Tris, pH 7.4, 137 mM NaCl, 2 mM EDTA, 10% glycerol, 1 mM PMSF, and 1× cOmplete™ protease inhibitor cocktail). For cytochrome c, COX IV, and GAPDH in either cytosolic or mitochondrial fraction, we obtained each fractionated sample using a commercially available kit (Mitochondria/Cytosol Fractionation Kit, BioVision, Catalog #K256-25, Milpitas, CA, USA).

To obtain cytosol samples of tissue, liver tissues were lysed with buffer consisting of follows; 0.1 mM EDTA, 10 mM HEPES (pH 7.8), 0.1 mM PMSF, 10 mM KCl, 1 mM DTT, 2 mM MgCl_2_, and 1250 μL protease inhibitor solution (Wako, Osaka, Japan). The homogenates were incubated (4 °C for 20 min), and then 10% NP-40, respectively. After centrifugation with the above lysis buffer (12,000 rpm at 4 °C for 2 min, Eppendorf 5415R, Hamburg, Germany). Then the supernatant was collected as a cytosol sample, and the lysates were suspended with 20 mL ice-cold lysis buffer for intending to extract nucleus (50 mM HEPES (pH 7.8), 50 mM KCl, 300 mM NaCl, 1 mM DTT, 0.1 mM EDTA, 1% (*v*/*v*) glycerol, 0.1 mM PMSF, and 100 μL protease inhibitor solution, respectively) and suspended and incubated (4 °C for 30 min), then performed centrifugation (12,000 rpm at 4 °C for 10 min). The supernatant was finally collected as a nuclear sample. Both cytosol and nuclear samples were stored at −80 °C for further experiments. 

Samples containing 10–12 μg of protein were electrophoresed through 8–15% SDS-PAGE and transferred to a nitrocellulose membrane. Each membrane was blocked with 5% (*w*/*v*) skim milk solution for 1 h and visualized using ECL reagents of GE Healthcare (Chicago, IL, USA). The bands were detected by Sensi-Q 2000 Chemidoc (Lugen Sci Co., Ltd., Gyeonggi-do, Bucheon-si, Korea). Antibodies used in western blotting are shown in Supplementary Figure S8. We followed the methods of Mi-Rae Shin et al. [14].

The quantification analysis of each band was analyzed by application of Image J 1.52 software (NIH, Bethesda, MD, USA).

### 2.10. Statistical Analysis 

All statistical data were expressed as mean ± SEM through the present manuscript by the performance of the Prism 9.2 software from GraphPad (La Jolla, CA, USA). Comparisons between two groups were performed using a two-tailed unpaired Student *t*-test. Comparisons for more than two groups, we performed one-way or two-way ANOVA followed by Tukey’s post-hoc tests.

## 3. Results

### 3.1. GF Attenuated TAA-Mediated Chronic Liver Injury

**Figure 1:**
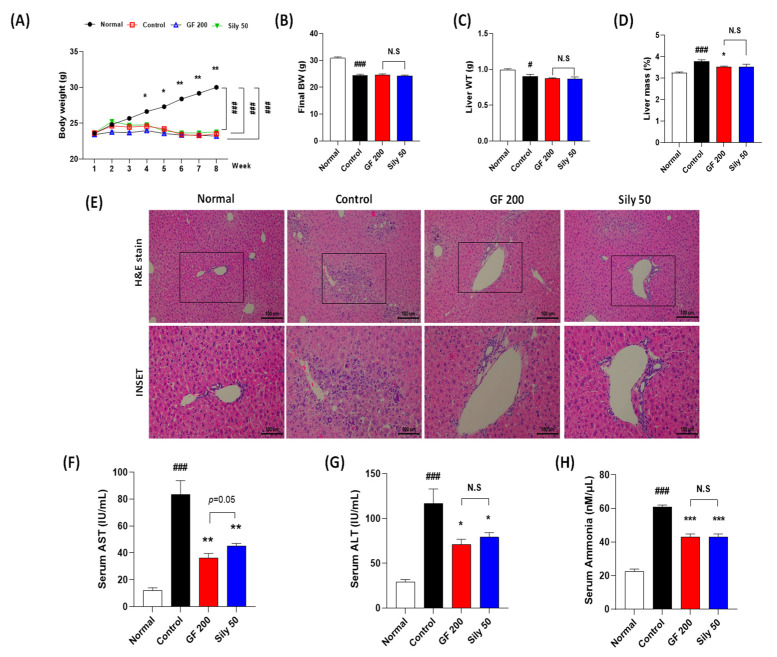
Effects of GF on the TAA-injected liver fibrosis of mice model. General outcomes for (**A**) body weight changes, (**B**) final body weight, (**C**) liver tissue weights, and (**D**) relative liver weights. (**E**) Histopathological examinations of H&E stain. (**F**) Serum AST, (**G**) ALT, and (**H**) ammonia levels. Data were expressed mean ± SEM (*n* = 9 for each group). N.S, not significant, ^#^ *p* < 0.05 and ^###^ *p* < 0.001 for Normal vs. Control; * *p* < 0.05, ** *p* < 0.01, and *** *p* < 0.001 for Control vs. GF 200 or Sily 50. H&E staining images were captured by light microscope condition (100- or 400-× magnifications).

TAA injection considerably decreased BW as compared with the normal group, and there were no significant differences among the control, GF 200, and Sily 50 groups during experiment periods. (*p* < 0.001 for time-dependent manners of BW changes and among the groups, Figure 1A). The final BW and liver WT were significantly reduced by TAA injection in the control group as compared with the normal group *(p* < 0.001 or 0.05 in Figure 1B,C), and both drug treatment groups didn’t show a significance as compared with the control group (Figure 1B,C). Relative liver mass was significantly increased by TAA injection in the control group as compared with the normal group (*p* < 0.001), whereas GF 200 significantly decreased relative liver tissue mass as compared with the control group, but not Sily 50 (*p* < 0.05, Figure 1D). Eight weeks of TAA injection severely led to liver damage by evidence of histopathological alterations based on the H&E staining. As compared to the normal group, the control group showed infiltrations of inflamed cells and hepatocyte cell deaths through the liver tissue (Figure 1E). Additionally, liver enzymes such as serum AST and ALT levels in the control group were significantly increased as compared with the normal group, respectively (Figure 1F,G). These liver injuries were well represented by abnormal elevations of serum ammonia levels in the control group (Figure 1H). Administration with GF during entire experimental periods expected not only attenuated liver inflamed cell infiltrations with hepatocyte deaths by histological inspection, but also significantly exerted to decrease serum AST, ALT, and ammonia levels, respectively (*p* < 0.01 for AST, *p* < 0.05 for ALT, and *p* < 0.001 for ammonia, Figure 1F–H). TAA induced hepatic tissue injuries were not only improved histopathological examinations in Sily 50 group (Figure 1E), but also significantly attenuated by features of serum biochemistries and serum ammonia levels (Figure 1F–H); however, silymarin 50 mg/kg treatment didn’t significantly alter general outcomes in the present study. 

Compared to the pharmacological properties between GF 200 and Sily 50, there was a tendency that GF 200 seemed better in serum AST and ALT levels than Sily 50, but other factors were shown similar effects. 

### 3.2. Effects of GF on TAA-Induced Hepatic Tissue Oxidation

**Figure 2:**
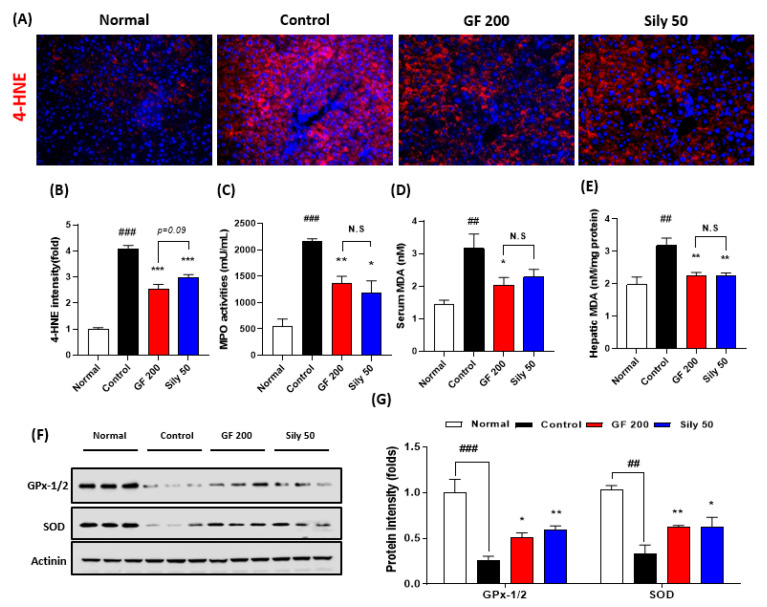
Antioxidant effects of GF on the TAA-induced hepatic tissue oxidation. (**A**) IF analysis against 4-HNE and (**B**) quantification analysis. (**C**) MPO activities in the serum level. (**D**) Serum MDA and (**E**) hepatic MDA levels. (**F**) Western blot analysis of GPx-1/2 and SOD in hepatic protein levels. (**G**) Quantification analysis of GPx-1/2 and SOD. Data were expressed mean ± SEM (*n* = 9 for each group). N.S, not significant, ^##^ *p* < 0.01 and ^###^ *p* < 0.001 for Normal vs. Control; * *p* < 0.05, ** *p* < 0.01, and *** *p* < 0.001 for Control vs. GF 200 or Sily 50. Images were captured by fluorescence filter equipped with microscope condition (100- or 400-× magnifications).

Next, we addressed that the TAA-induced hepatic injury in the present study by focusing on the oxidative stress and antioxidant components alterations. First, we measured IF analysis against 4-HNE which is a correlation marker of final oxidative stress product (*p* < 0.001). As expected, the control group significantly increased the positive signals of 4-HNE as compared with the normal group. Additionally, serum MPO activities in the control group were significantly increased as compared with the normal group (*p* < 0.001). This pathological alteration was well supported by abnormal oxidative stress status in both serum and hepatic protein levels of MDA, which is a marker of lipid peroxidation (*p* < 0.01, Figure 2D,E), whereas administration with GF 200 mg/kg significantly ameliorated the above pathological statuses (*p* < 0.001 for 4-HNE IF analysis, *p* < 0.01 for MPO and hepatic MDA, and *p* < 0.05 for serum MDA, Figure 2A–E). Western blot analysis well displayed that the hepatic antioxidants such as GPx-1/2 and SOD were significantly deterred by 8 weeks of TAA injection in the control group (*p* < 0.001 or 0.01), whereas GF 200 significantly prevented from deterioration of two major antioxidant enzymes in the hepatic tissue against TAA mediated-hepatic tissue oxidation (*p* < 0.05 or 0.01 in Figure 2F,G). 

Sily 50 group exhibited similar efficacies of GF 200 as compared with the control group against hepatic oxidations (*p* < 0.05, 0.01, or 0.001, Figure 2A–G).

### 3.3. Effects of GF on Hepatic Inflammation

**Figure 3:**
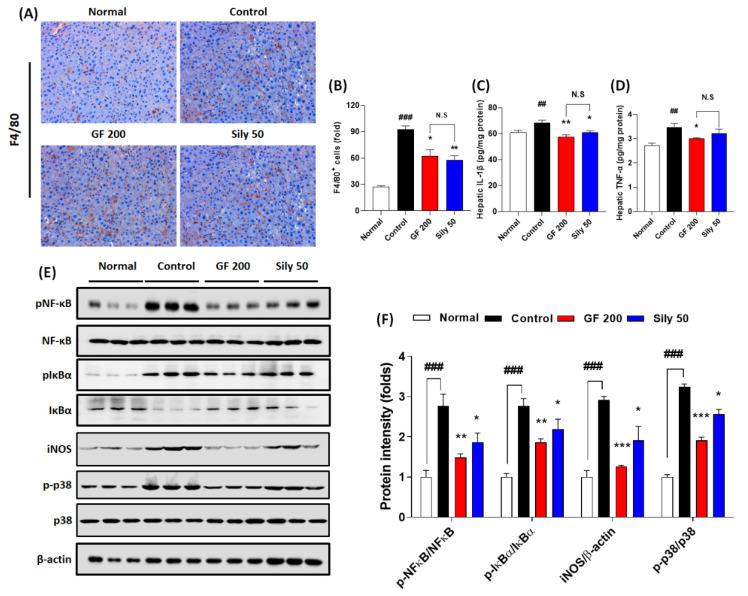
Effects of GF on TAA-injected hepatic inflammation. (**A**) IHC analysis against F4/80 and (**B**) quantification analysis. (**C**) Hepatic protein levels of IL-1β and (**D**) TNF-α. (**E**) Western blot analysis of inflammatory related proteins including p-NF-κB, NF-κB, p-IκBα, IκBα, iNOS, p-p38, and p38 and (**F**) protein intensity. Data were expressed mean ± SEM (*n* = 4 for each group in IHC analysis; n = 9 for each group for IL-1β and TNF-α; *n* = 3 for each group in Western blot analysis). N.S, not significant, ^##^ *p* < 0.01 and ^###^ *p* < 0.001 for Normal vs. Control; * *p* < 0.05, **p < 0.01, and *** *p* < 0.001 for Control vs. GF 200 or Sily 50. H&E staining images were captured by light microscope condition (400-× magnifications).

Hepatic inflammation is one of the representative pathological phenotypes of TAA-induced hepatic injury. Thus, we examined the pharmacological properties of GF focusing on the inflammatory reaction-related markers. We presented IHC against F4/80 which is a marker of Kupffer cells in the liver tissue resided macrophage. As we expected, the positive signals were remarkably enhanced in the control group as compared with the normal group (*p* < 0.001), while GF 200 significantly attenuated these positive signals as compared with the control group (*p* < 0.05 in Figure 3A,B). Pro-inflammatory cytokines such as IL-1β and TNF-α in hepatic protein levels of the control groups were considerably increased in the response to TAA-induced hepatic injury (*p* < 0.01); however, administration with GF 200 mg/kg exerted to decrease these pro-inflammatory cytokines as compared with the control group (*p* < 0.05 or 0.01 in Figure 3C,D). To elucidate the pharmacological properties of GF 200 focusing on the possible molecular signaling pathways of hepatic inflammation, we performed Western blot analysis against NF-κB, IκBα, iNOS, and p38 with their phosphorylated forms in hepatic protein levels, all targeted proteins in the control group were drastically altered according to our expectation as compared to the normal group (*p* < 0.001); however, we also observed that GF 200 mostly normalized the above inflammatory target proteins’ abnormalities with statistical significance as compared with the control group (*p* < 0.01, or 0.001 in Figure 3E,F).

Administration with Silymarin 50 mg/kg showed beneficial effects on F4/80 IHC analysis (*p* < 0.01), hepatic protein levels of IL-1β (*p* < 0.05), and phosphorated forms of NF-κB, IκBα, and p38 and iNOS protein levels which were evidenced by Western blot analysis (*p* < 0.05 for Figure 3E,F). 

### 3.4. Effects of GF on the Hepatic Fibrosis

**Figure 4:**
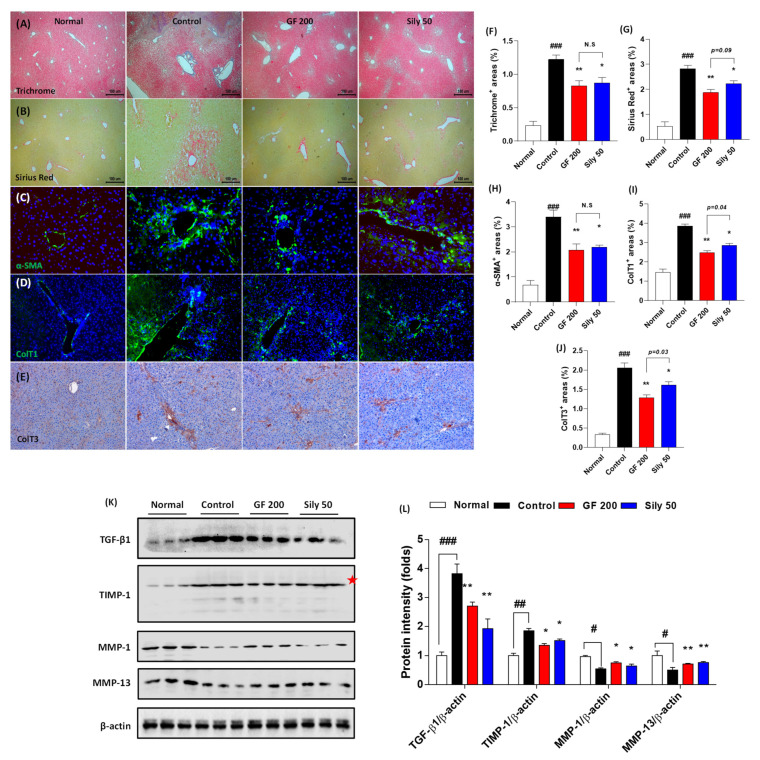
GF attenuates liver fibrosis by degradations of ECMs in tissue. (**A**) Masson’s trichrome staining, (**B**) Sirius Red staining, (**C**) IF analysis against α-SMA, (**D**) Collagen type 1, and (**E**) IHC against Collagen type 3. (**F**–**J**) Quantification analyses for Masson’s trichrome staining, Sirius Red staining, IF analysis against α-SMA, Collagen type 1, and IHC against Collagen type 3. (**K**) Western blot analysis of ECM proteins TGF-β1, TIMP-1, MMP-1, and MMP-13 and (**L**) protein intensity. Data were expressed mean ± SEM (*n* = 4 for each group in staining images; *n* = 3 for each group for Western blot analysis). N.S, not significant, ^#^ *p* < 0.05, ^##^ *p* < 0.01, and ^###^ *p* < 0.001 for Normal vs. Control; * *p* < 0.05 and ** *p* < 0.01 for Control vs. GF 200 or Sily 50. Masson’s trichrome, Sirius Red staining, and IHC against Collagen type 3 images were captured by light microscope condition (40-,100-, and 200-× magnifications). IF images were observed by fluorescence filtered equipped microscopy condition (200-× magnifications).

Next, we further examined the pharmacological effects of GF on hepatic fibrosis. Both Masson’s trichrome and Sirius Red stains well evidenced that TAA-induced liver fibrosis by huge amount collagen deposition through hepatic tissue (positive color for blue in Masson’s trichrome and red color for Sirius Red staining) in the control group as compared with the normal group (*p* < 0.001). Representative ECMs molecules of IF or IHC against α-SMA, Collagen type 1, and Collagen type 3 were also abnormally enhanced through liver tissue by TAA injection (*p* < 0.001). These alterations, as our expectation, were significantly resolved in the GF 200 group as compared to the control group (*p* < 0.01 in Figure 4A–J). These alterations were well correlated with the numbers of HSCs in the liver tissue which were by the performance of IF analysis against Desmin. Desmin positive signals (part of red fluorescence) were remarkably enhanced in the control group as compared with the normal group, but these signals were drastically decreased as compared with the control group (Supplementary Figure S3). Additionally, the representative profibrogenic cytokine and ECMs-related target proteins including TGF-β1, TIMP-1, MMP-1, and MMP-13 in the hepatic protein levels were abnormally altered in the control group as compared with the normal group (*p* < 0.05, 0.01, or 0.001, respectively). Administration with GF 200 mg/kg not only significantly led to a decrease in TGF-β1 and TIMP-1, but also increase MMP-1 and MMP-13 as compared to the control group (*p* < 0.05 or 0.01 in Figure 4K,L). Interestingly, we also examined the ductular reactions of bile duct area through the liver tissue, but there were no significant features, only the number of bile duct area were increased in the control group as compared with the normal group. In addition, no differences were not observed between GF 200 and Sily 50 as compared with the control group by evidence of IHC analysis against CK-19 (Supplementary Figure S4). 

The positive control group, Sily 50, showed similar properties on the liver fibrosis by amelioration collagen accumulation as well as exertion of fibrogenic cytokine and ECMs-related proteins (*p* < 0.05 or 0.01). The number of HSCs through the liver tissue was diminished in Sily 50 as compared with the control group (Supplementary Figure S3), but Sily 50 didn’t alter ductular reactions (Supplementary Figure S4). 

### 3.5. Pharmacological Properties of GF against TAA-Induced Hepatic Fibrosis via Modulations of SIRT1

**Figure 5:**
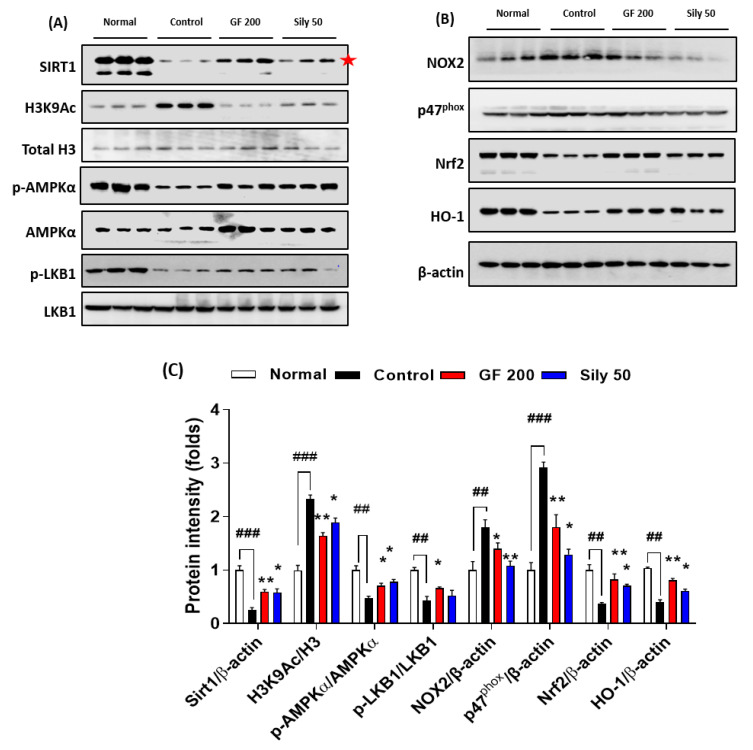
GF ameliorates liver fibrosis via regulations of AMPK/SIRT1 signaling pathways. Western blot analysis of SIRT1 and its deacetylate target proteins such as H3K9Ac, Total H3, p-AMPKα, and AMPKα, p-LKB1, and LKB1 (**A**), and NOX2, p47^phox^, Nrf2, and HO-1 (**B**). (**C**) Quantification analysis of Western blot analysis. Data were expressed mean ± SEM (*n* = 3 for each group for Western blot analysis). ^##^ *p* < 0.01 and ^###^ *p* < 0.001 for Normal vs. Control; * *p* < 0.05 and ** *p* < 0.01 for Control vs. GF 200 or Sily 50.

To validate the possible underlying mechanism of GF on TAA-injected hepatic fibrosis, we focused on the epigenetic regulator, SIRT1 which is well known for a NAD^+^, NADH-dependent class III protein deacetylase-enzyme. Our Western blot analysis results well evidenced that SIRT1 in hepatic protein levels of the control group was considerably depleted as compared with the normal group (*p* < 0.001), and H3K9Ac also well support the above alterations in hepatic protein levels of TAA-injected liver tissue by drastically increase manners compared to the normal group (*p* < 0.001). Administration with GF 200 mg/kg significantly normalized against these alterations in hepatic protein levels as compared with the control group (*p* < 0.01 for SIRT1 and H3K9Ac). Additionally, the up-streaming molecule of AMPKα, which is a p-LKB1 was significantly decreased in the control group as compared with the normal group, the GF significantly increased p-LKB1 as compared with the control group (*p* < 0.05). Regarding the molecular levels of the relationship between SIRT1 and AMPKα, we observed that AMPKα was drastically activated by the deterioration of SIRT1 (*p* < 0.05). We further observed that SIRT1 target oxidative stress-related proteins such as NOX2 and p47^phox^ were significantly normalized by GF 200 against TAA-injected hepatic fibrosis (*p* < 0.05 or 0.01). Additionally, AMPKα targeted proteins which are NADPH activity relying on antioxidant enzymes Nrf2 and HO-1 were also deterred by TAA and remarkably recovered by GF 200 mg/kg (*p* < 0.01, Figure 5A–C).

As a positive drug treatments Silymarin 50 mg/kg, also well showed beneficial properties on TAA-induced liver fibrosis by controlling of SIRT1/AMPKα signaling pathways with statistical significances as compared with the control group (*p* < 0.01, Figure 5A–C).

### 3.6. GF Prevents Hepatocyte Oxidation by Increase of SIRT1 in Time Dependent Manners

**Figure 6:**
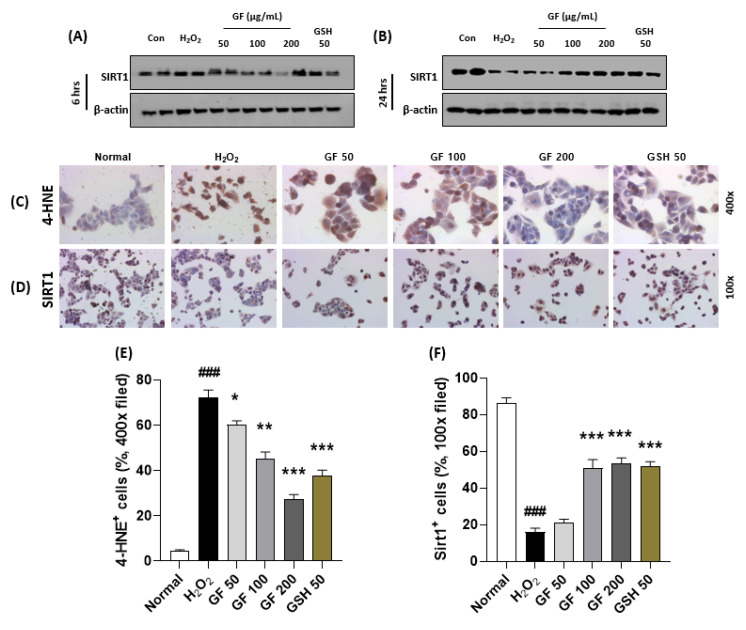
GF attenuates hepatocyte oxidative stress via prevention from SIRT1 depletions. Western blot analysis in H_2_O_2_ (500 µM) treated HepG2 cells for 6 h or 24 h with or without GF (50, 100, 200 µg/mL). Cellular protein levels of SIRT1 for 6 h (**A**) and 24 h (**B**). IHC analysis against 4-HNE (**C**) and SIRT1 (**D**). Quantification analysis of 4-HNE (**E**) and SIRT1 (**F**). Data were expressed mean ± SEM (*n* = 4 for IHC analysis). ^###^ *p* < 0.001 for Normal vs. H_2_O_2_ and * *p* < 0.05, ** *p* < 0.01, and *** *p* < 0.001 for H_2_O_2_ vs. GF or GSH. IHC images were captured by microscope condition (100- or 400-× magnifications).

To explain the pharmacological mechanisms of GF 200 on oxidative stress-induced hepatocyte damage, we explored hydrogen peroxide (H_2_O_2_)-induced hepatocyte oxidation using HepG2 cells which is a human blastoma cell line. First, we intended to confirm the alterations of SIRT1 would be affected by time-dependent manner or not. Interestingly, we observed that chronic exposure of H_2_O_2_ (500 µM incubation for 24 h, Figure 6B) in the HepG2 cells were considerably depleted SIRT1 in the protein levels as compared with the control group, but not acute exposure (6 h, Figure 6A). As expected, we also confirmed that chronic exposures to H_2_O_2_ could significantly enhance dead cell numbers (*p* < 0.001, Supplementary Figure S5A,B), which were well supported by abnormal releases of cytochrome *c* from mitochondria to cytosolic levels by evidence of Western blot analysis (Supplementary Figure S5C,D). To verify oxidative stress is a deciding factor by SIRT1 existence or not, we further performed IHC analysis against 4-HNE and SIRT1 after 24 h of H_2_O_2_ incubated cells. 4-HNE positive signals were also hugely enhanced in H_2_O_2_ group as compared with the normal group (*p* < 0.001 in Figure 6C,E), which were reversely appeared in the positive signals of SIRT1 in the drug-treated groups (*p* < 0.001 in Figure 6D,F), respectively.

Pre-treatment with GF with various doses (6 h prior to hydrogen peroxide with 50, 100, and 200 µg/mL) significantly prevented the depletion of SIRT1 in cellular protein levels of the chronic exposure condition (*p* < 0.001 for Figure 6B,D,F). According to these properties, GF could block oxidative stress as well as cell death which were well evidenced by 4-HNE (*p* < 0.001 for Figure 6C,E), LIVE/DEAD cell (*p* < 0.001 for Supplementary Figure S5A,B), Western blot analysis of cytochrome *c* in mitochondria and cytosolic levels (Supplementary Figure S5C,D). 

### 3.7. GF Promotes Inactivation of TGF-β1 Incubated Culture-Induced Activated HSCs by Prevention of SIRT1 

**Figure 7:**
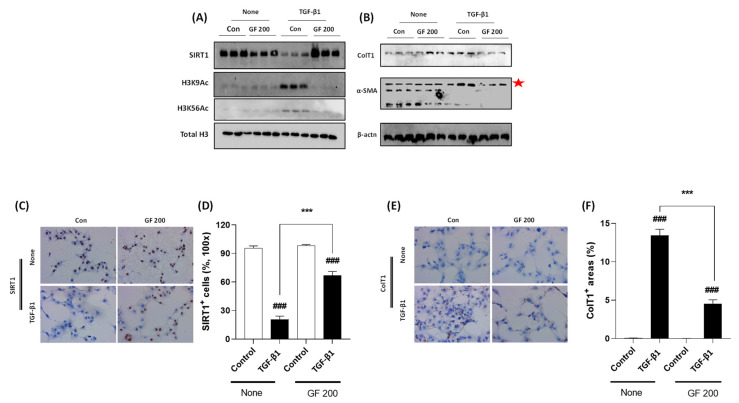
GF Alleviates TGF-β1-induced LX-2 Cell Activation via Enhancement of SIRT1. Western blot analysis in TGF-β1 treated LX-2 cells with or without GF. (**A**) Cellular protein levels of SIRT1, H3K9Ac, H3K56Ac, and Total H3. (**B**) Cellular protein levels of Collagen type 1, α-SMA, and β-actin. IHC analysis against SIRT1 (**C**) and its quantification analysis (**D**), and Collagen type 1 IHC (**E**) and its quantification analysis (**F**). Data were expressed mean ± SEM (*n* = 3 for Western blot analysis, *n* = 4 for IHC analysis). ^###^ *p* < 0.001 for Control vs. TGF-β1 and *** *p* < 0.001 for None vs. GF 200. IHC images were captured by microscope condition (100× magnifications).

To validate the underlying mechanisms of GF against liver fibrosis, we performed TGF-β1 treated LX-2 cell activation in vitro experiment. As our expectation, TGF-β1 considerably decreased cellular protein levels of SIRT1 which was well supported by Western blot analysis of H3K9Ac and H3K56Ac. Cellular protein levels of both Collagen type 1 and α-SMA were drastically increased by TGF-β1, whereas pre-treatment with GF considerably decreased the above alterations by reverse manners of SIRT1 in protein levels (Figure 7A,B). Accordingly, the Western blot analysis results and IHC analysis well supported the effects of GF by regulations of SIRT1 with statistical significances (Figure 7C–F). 

## 4. Discussion

Liver fibrosis is a process of wound and healing under the pathological status of chronic liver diseases. In liver fibrosis, the HSCs are well known to target cell type in the liver tissue to decide progression the next step of liver disease called liver cirrhosis or reversible to the non-pathological status of liver diseases [8,15]; however, there is no therapeutics to cure the liver fibrosis globally till nowadays. Thus, we aimed to investigate to develop medicine to treat liver fibrosis based on the natural plant, GF, especially [16] (Figure 1E–H), but didn’t significantly recover in general physiological outcomes (Figure 1A–D). 

Accumulated documents well displayed that TAA is a toxic agent to induce liver diseases via abnormal modulations of liver detoxification enzymes which can evoke oxidative stress-induced liver tissue damage and progress severe stages of liver diseases, called liver fibrosis and cirrhosis [17,18,19]. Thus, we addressed the effects of GF on the TAA-injected hepatic tissue oxidation. As expected, GF not only led to decreases of 4-HNE formations, serum and hepatic tissue levels of MDA, and serum MPO activities (Figure 2A–E) but also prevented hepatic endogenous antioxidants depletions such as SOD and GPx-1/2, respectively (Figure 2F,G). Additionally, chronic liver inflammation is invariably connected to liver fibrosis [20,21], and we partially proved the anti-inflammatory activities of GF against TAA-induced liver tissues. As shown in Figure 3A,B, the numbers of Kupffer cells in the hepatic tissue were considerably decreased by GF and pro-inflammatory such as IL-1β and TNF-α which are mainly activated by NF-κB in activated Kupffer cells during hepatic inflammation (Figure 3C,D). Furthermore, proteins by pending of NF-κB signaling pathway were normalized by GF (Figure 3E,F). Our findings are well comprised of the representative pathophysiological status of hepatic inflammation by TAA-induced hepatic liver injury [22,23] and sufficient to support anti-hepatic inflammation effects on liver fibrosis by attenuations of pro-inflammatory cytokines as well as Kupffer cells activation related molecules (Figure 3A–F).

As we above mentioned, finally our mice were led to liver fibrosis conditions that mean excessive accumulation of ECMs proteins including predominantly fibrillar Collagen type 1 or Collagen type 3, and α-SMA, respectively [2,15]. Thus, we next investigated that the effects of GF on the modulations of activated HSCs in the hepatic tissue. Regarding liver fibrosis, our first finding which is IF analysis against HSCs by stating of HSCs were well corresponded to the GF effects by reducing their numbers in liver tissues (Supplementary Figure S3). Next, we further confirmed that anti-hepatofibrotic effects of GF are mainly attributed to alleviations of the huge amount of ECMs accumulation in liver tissues by evidence of both Masson’s trichrome and Sirius Red stains (Figure 4A,B,F,G). These results are well supported by degradations of ECMs including α-SMA, Collagen type 1, and Collagen type 3, respectively (Figure 4C–E,H–J). Hepatic protein levels of TGF-β1, known for pro-fibrogenic cytokine to promote HSCs activation or proliferation were drastically normalized by GF and other proteins such as TIMP-1, MMP-1, and MMP-13 which are known as ECMs promoters or degradations as responses of liver fibrosis [24,25]. Previous studies well reported that bile duct cells proliferation or ductular reactions in the liver tissue by TAA-induced liver fibrosis model [26]; however, in the present study ductular reactions in hepatic tissues were not affected by treatment with either TAA or GF (Supplementary Figure S4). 

Liver fibrosis is normally comprehended by complicated events from various liver tissue-specific cell types including hepatocyte, Kupffer cells, and HSCs, respectively. Therefore, it is needed to understand the whole liver tissue events with specific molecular signaling transduction pathways. On the other hand, SIRT1 that is an epigenetic regulator well known to NAD^+^-dependent histone deacetylase is deeply implicated in liver diseases by modulations of redox status, inflammation, and cell death, respectively [27,28,29,30,31]; however, its roles in liver fibrosis is not clearly studied yet. Thus, we focused on our liver tissues by application of Western blot analysis and obtained that GF apparently inhibited H3K9Ac by blocking SIRT1 depletion in the liver protein levels (Figure 5A,C). As a partnership with SIRT1, AMPK is applied to regulate energy metabolism especially various energy homeostasis imbalances provoked pathological conditions [32]. This signaling pathway is a crucial factor in the approaches of various liver diseases [33,34,35]. The up-stream molecule of AMPKα, which is known to LKB1 is deeply associated with liver inflammation and metabolism [36,37]. According to our expectation, GF not only drastically increase AMPKα in liver protein levels, but also normalized hepatic protein levels of AMPK/SIRT1 signaling related or target molecules including NOX2, p47^phox^, Nrf2, and HO-1, which mainly belong to liver tissue oxidation were significantly normalized by GF, especially respectively (Figure 5A,C). 

Since we revealed that GF showed its pharmacological properties via regulation of hepatic SIRT1 and its related proteins, we further validated its pharmacological functions of GF in liver cell type-specific manners using HepG2 cells and LX-2 cells which are well corresponded to the hepatocytes and HSCs, respectively. First, we verified chronic oxidative stress depleted hepatocyte cellular levels of SIRT1 in H_2_O_2_ incubation HepG2 cells of 24 h experiment, not 6 h by applications of Western blot analysis and IHC analysis (*p* < 0.001 in Figure 6A,B,D,E). The IHC against 4-HNE analysis well supported the above results (*p* < 0.001 in Figure 6C,E). Pre-treatment with GF with various doses significantly prevented the depletion of SIRT1 in cellular protein levels of the chronic exposure against H_2_O_2_ condition (*p* < 0.001). Moreover, GF could block oxidative stress by the suppression of 4-HNE (*p* < 0.001). Along with this finding, the cell death signals were also normalized by GF by relieving apoptosis-related proteins, especially cytochrome *c* (*p* < 0.001 in Supplementary Figure S5A–D). In the case of HSCs, we can explain GF efficiently worked decreases of ECMs including Collagen type 1, 3, and α-SMA by prevention of SIRT1 deteriorations in TGF-β1 treated activated HSCs (*p* < 0.001 in Figure 7A–F and Supplementary Figure S6). 

Previous studies well reported that geniposide which is an active compound from GF in various liver diseases especially NASH using pre-clinical in vivo models [38,39] and it also exerted to inhibit activations of HSCs [40], respectively. Pathophysiological progression of liver fibrosis, HSCs are the main target of advance this disease or reversely regression to the normal status [41]. Furthermore, GF also exerted antioxidant effects against ischemia/reperfusion in liver tissue of mice model [42] and alcoholic liver injury. 

Unfortunately, till nowadays, there is no therapeutic access to treat liver fibrosis in the clinical area, so it is urgently sounded to develop efficacy guaranteed drugs based on the explanations of underlying mechanisms. Based on the accumulations of numerous clinical practice experiences, there are many possible candidates using herbal drugs to treat liver fibrosis [43,44,45]. Additionally, it is also needed to consider a possible mechanism to treat liver fibrosis based on the clear molecular levels of interactions. According to this, we focused on the well-known epigenetic regulator, SIRT1, which is histone deacetylase targeted to HSCs activation, hepatocyte oxidation, and inflammation, respectively [46,47]. 

In the present manuscript, we proved GF exerted to attenuate hepatic fibrosis against TAA-induced mice model by reduction of hepatocyte oxidation, amelioration of hepatic inflammation, decreases of ECM deposition through the liver tissue during fibrosis progression. Regarding the pharmacological effects of GF, we can conclude its actions as follows; (1) GF showed strong antioxidant effects by enhancement of endogenous antioxidant components in the liver tissue especially recoveries of SOD and GPx. (2) GF could regulate Kupffer cell activation and their numbers by diminishing pro-inflammatory cytokines. (3) GF strongly led to inactivate HSCs activation by evidence of amelioration of ECM resolutions and pro-fibrogenic cytokines during liver fibrosis. The corresponded mechanisms for the above properties of GF may regulate AMPK/SIRT1 signaling pathway in hepatocytes and HSCs, especially focusing on the beneficial effects of SIRT1 in both cell types. 

Liver fibrosis is very a critical stage in the progression, which can decide to reserve normal stage or its advanced pathological stage such as liver cirrhosis or HCC, respectively. Nevertheless many trials to develop anti-hepatofibrosis therapeutics, there is no effective way existed in the world. From our study, we believe that GF would be one of the potential drug candidates to care for liver fibrosis. Therefore, further studies would be acquired to prove the safety and toxicity for clinical uses in the upcoming future. 

## Data Availability

The data presented in this study are available on request from the corresponding author.

## Supplementary Materials

The following data are available online at https://www.mdpi.com/article/10.3390/antiox10111837/s1, Figure S1: Chemical composition analysis of Geniposide; Figure S2: Experimental scheme for animal experiment; Figure S3: Representative immunofluorescence image against Desmin; Figure S4: Representative images of bile duct reactions; Figure S5: Effects of Gardeniae Fructus against hepatocyte oxidation-induced cellular apoptosis; Figure S6: Effects of Gardeniae Fructus against LX-2 cell activation; Figure S7: Antibody lists for Western blot analysis; Figure S8: Antibody lists for Immunohistochemistry and immunofluorescence analysis.
