# Supplementary figures and images for: Gardeniae Fructus Attenuates Thioacetamide-Induced Liver Fibrosis in Mice via Both AMPK/SIRT1/NF-κB Pathway and Nrf2 Signaling
Source: Antioxidants (Basel). 2021 Nov 19;10(11):1837. doi: 10.3390/antiox10111837 (PMC8614944; doi:10.3390/antiox10111837)

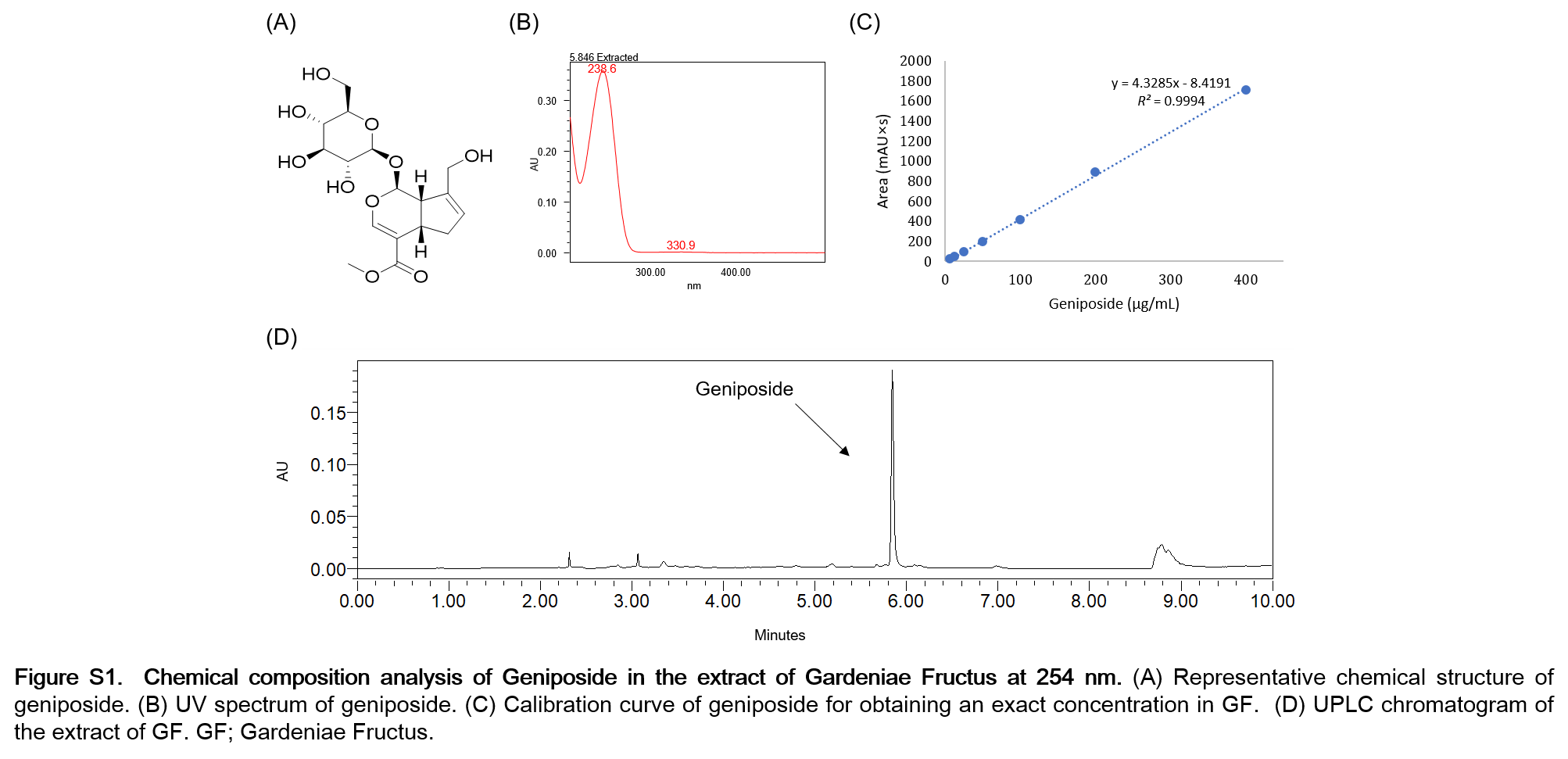

Supplement: Supplementary file 1 [file antioxidants-10-01837-s001.zip › Figure S1.tif]

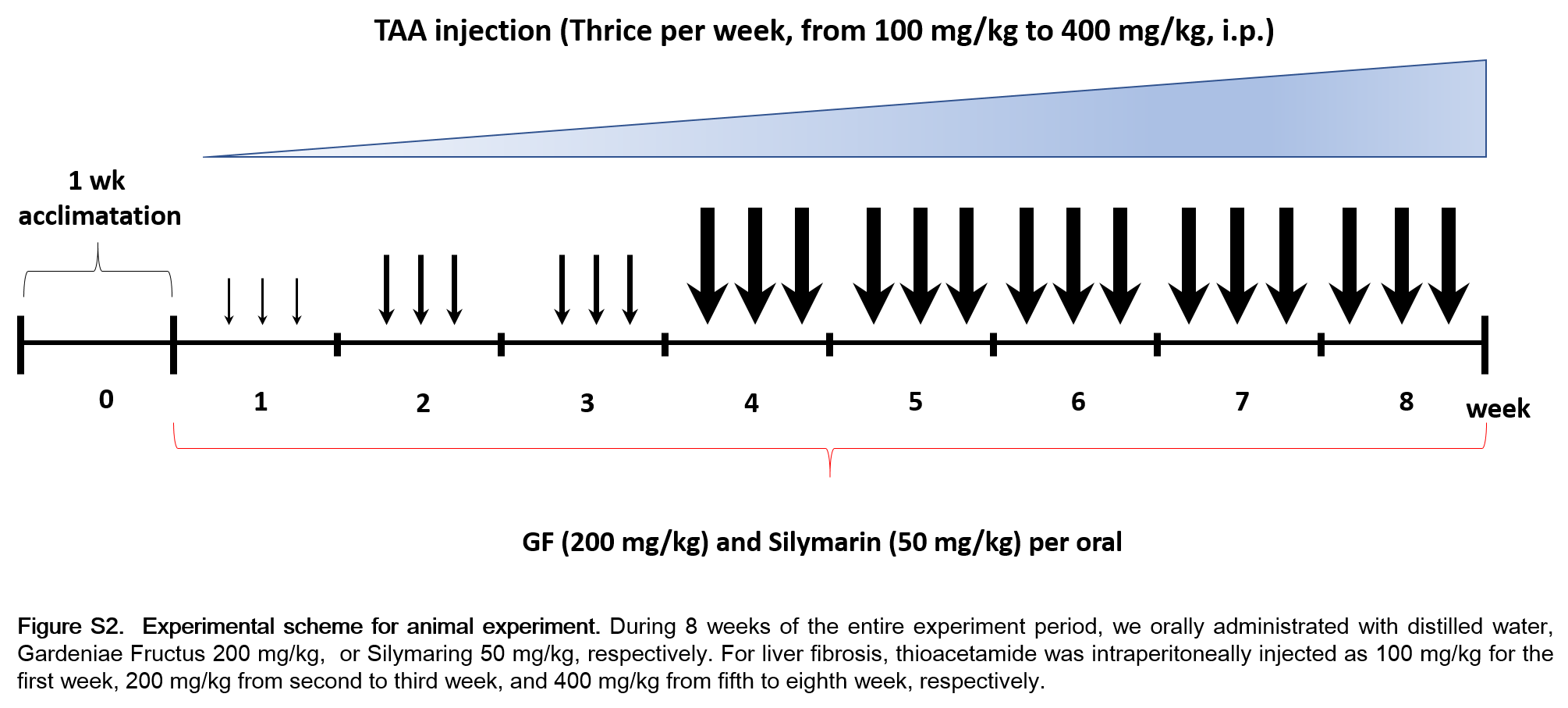

Supplement: Supplementary file 1 [file antioxidants-10-01837-s001.zip › Figure S2.tif]

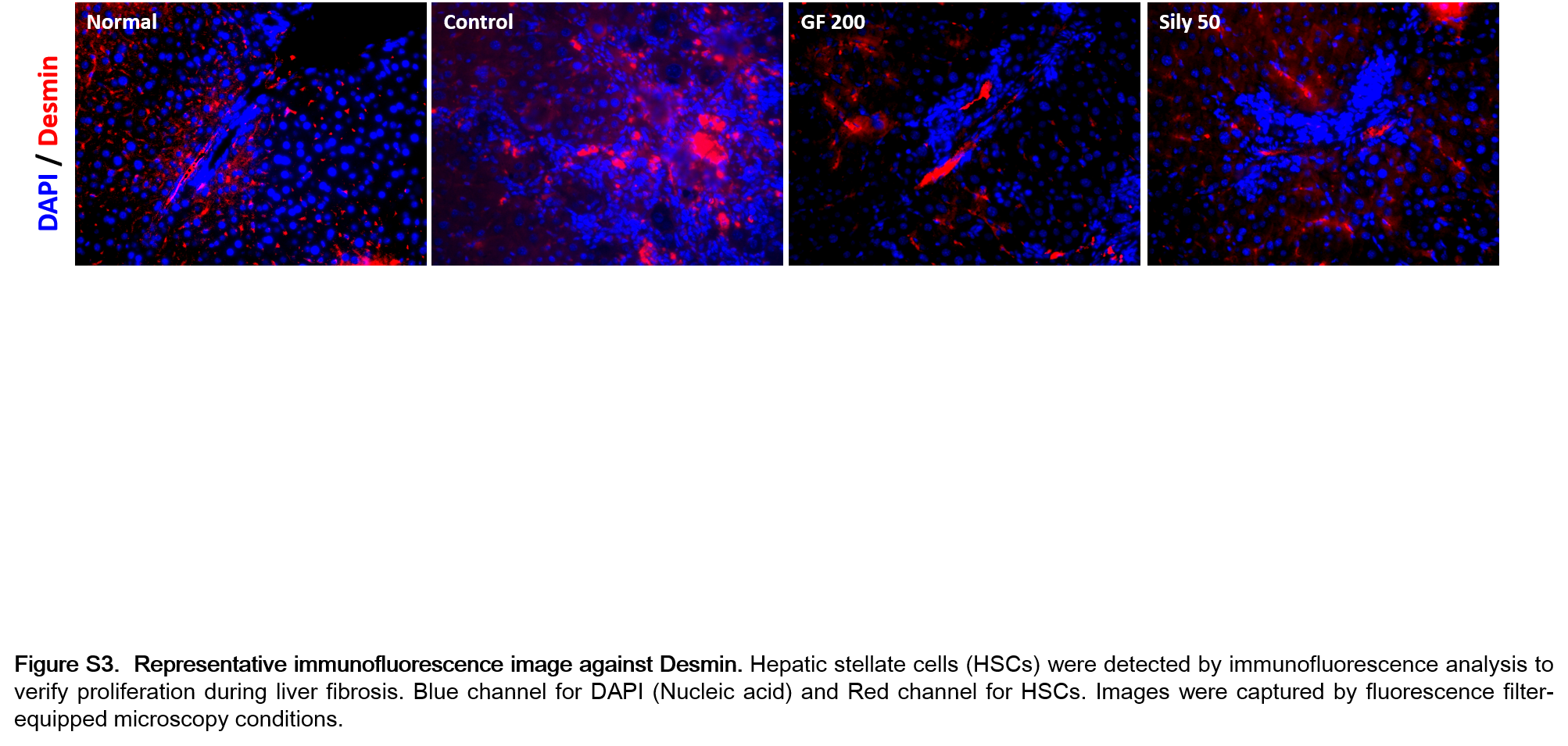

Supplement: Supplementary file 1 [file antioxidants-10-01837-s001.zip › Figure S3.tif]

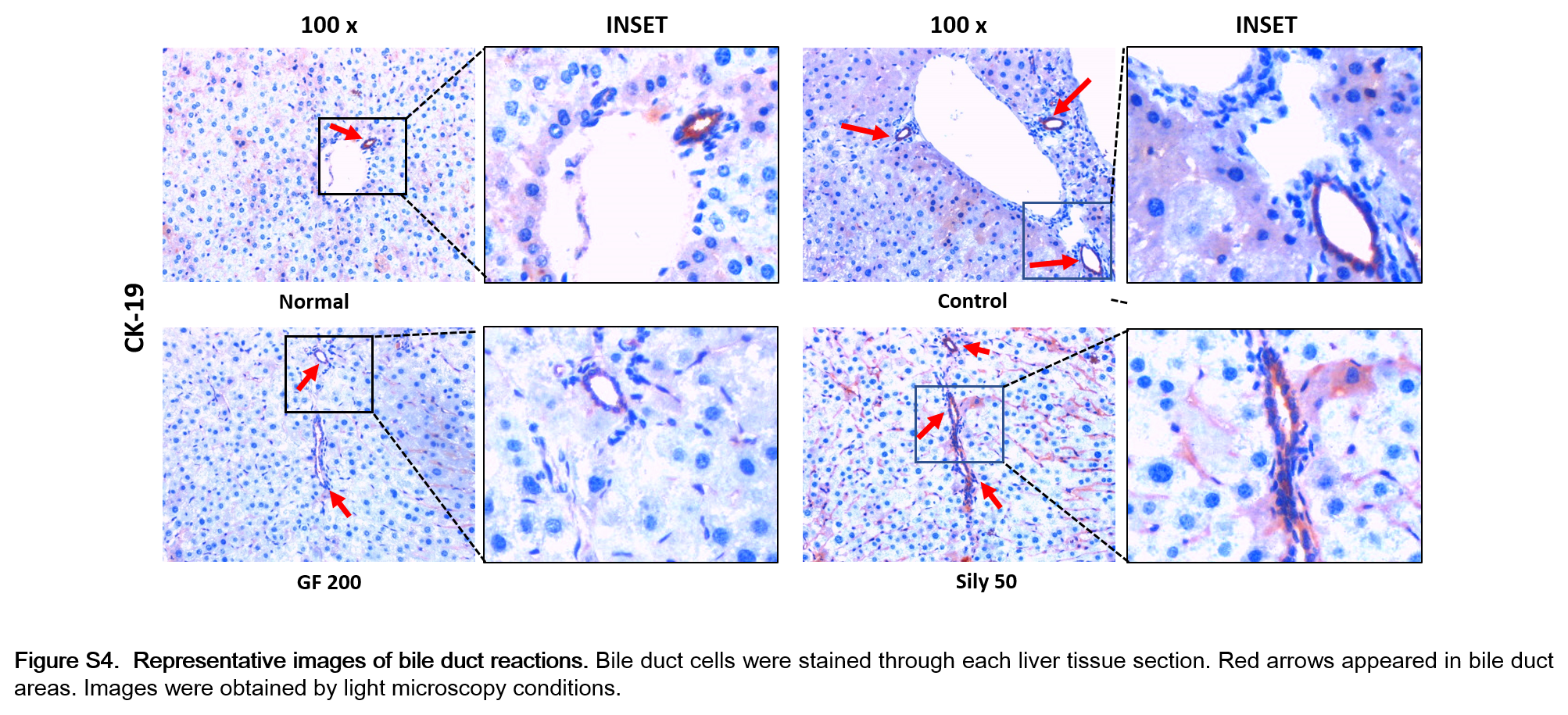

Supplement: Supplementary file 1 [file antioxidants-10-01837-s001.zip › Figure S4.tif]

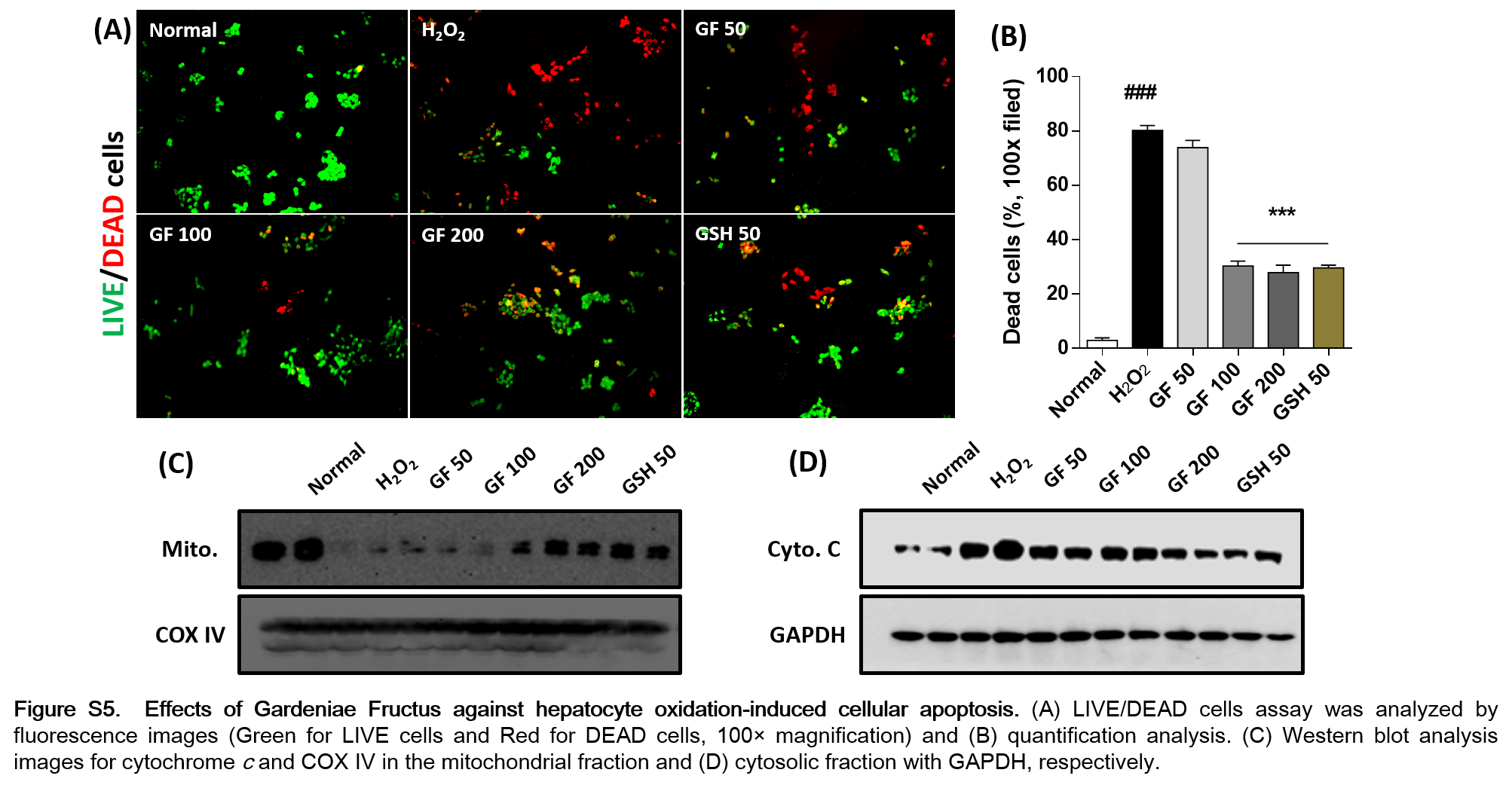

Supplement: Supplementary file 1 [file antioxidants-10-01837-s001.zip › Figure S5.tif]

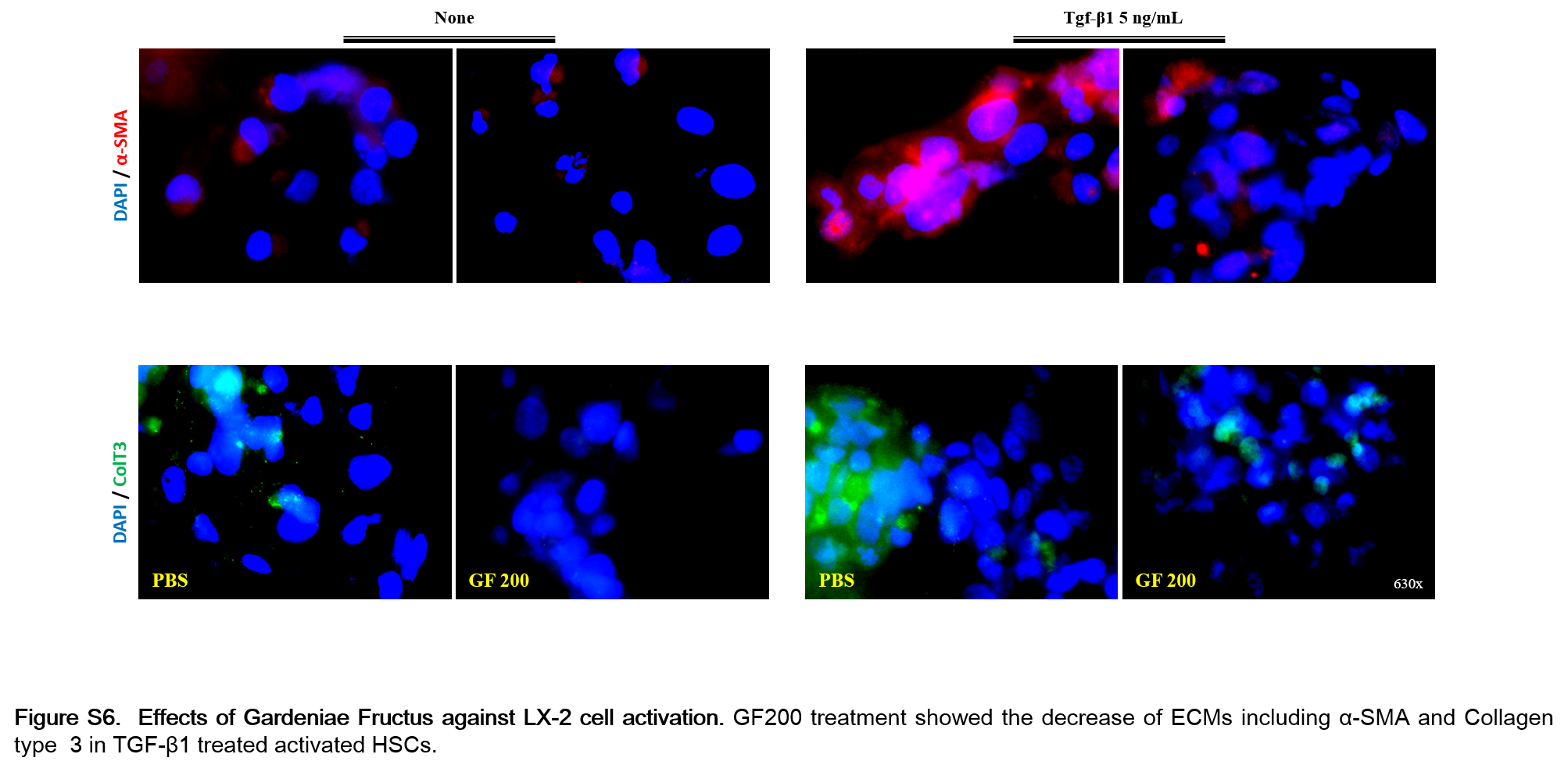

Supplement: Supplementary file 1 [file antioxidants-10-01837-s001.zip › Figure S6.tif]

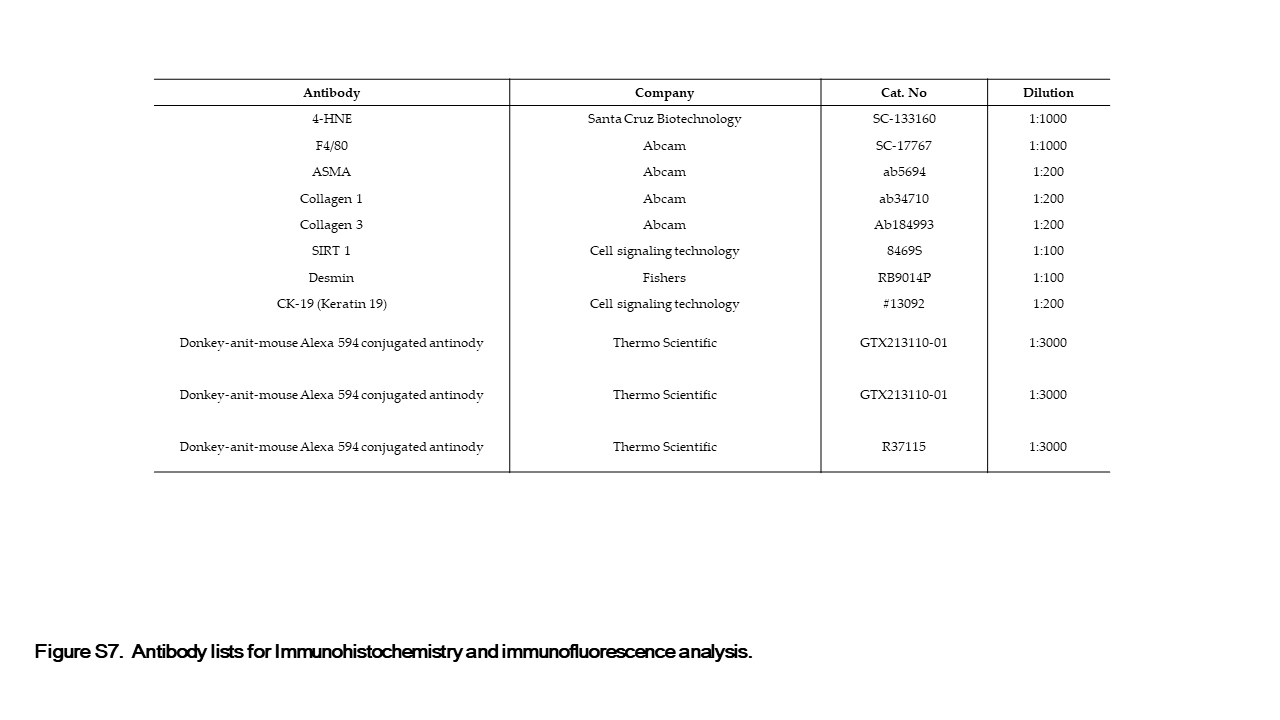

Supplement: Supplementary file 1 [file antioxidants-10-01837-s001.zip › Figure S7.TIF]

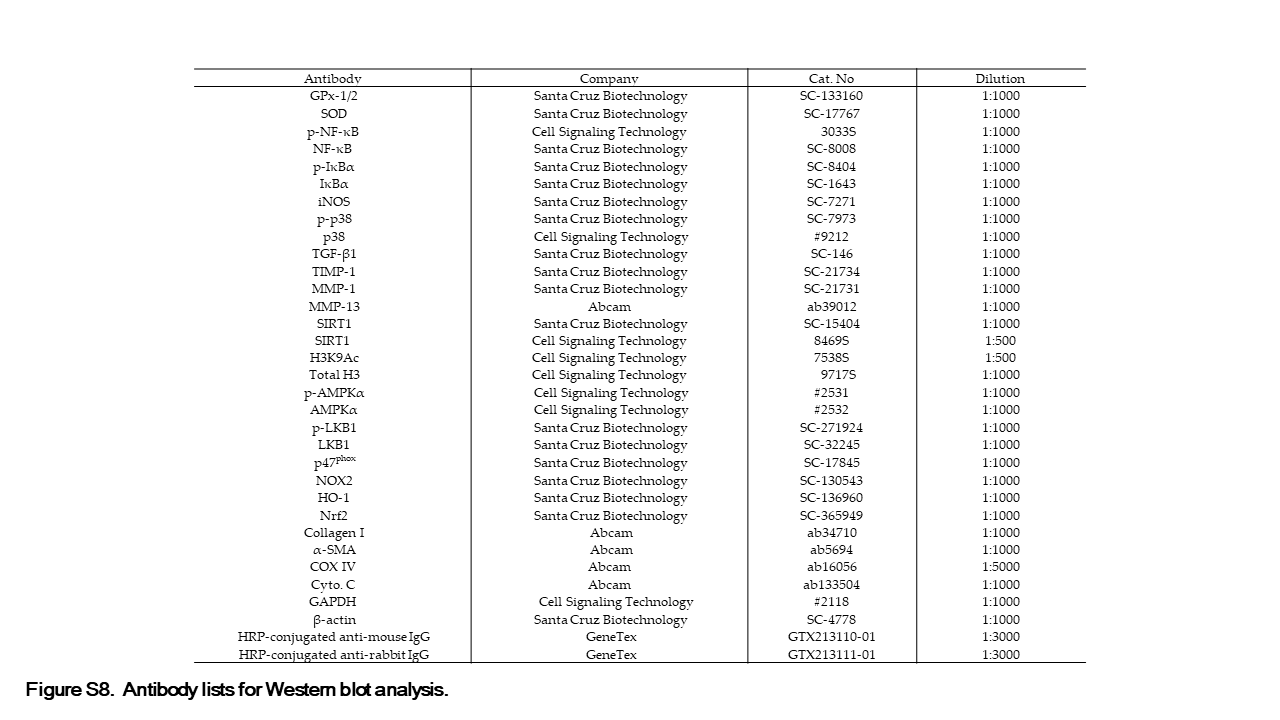

Supplement: Supplementary file 1 [file antioxidants-10-01837-s001.zip › Figure S8.TIF]
